# Platelet-to-Monocyte Ratio as a Novel Promising Agent for the Prognosis of Hepatitis B Virus-Associated Decompensated Cirrhosis

**DOI:** 10.1155/2023/6646156

**Published:** 2023-07-14

**Authors:** Jun Zhou, Xin Li, Min Wang, Chunrong Gu, Jingping Liu

**Affiliations:** ^1^Department of Laboratory Medicine, The First Affiliated Hospital of Nanjing Medical University, Nanjing, Jiangsu, China; ^2^Branch of National Clinical Research Center for Laboratory Medicine, Nanjing, Jiangsu, China; ^3^Department of Laboratory Medicine, The Affiliated Taizhou People's Hospital of Nanjing Medical University, Taizhou, Jiangsu, China

## Abstract

**Aim:**

The present study aimed at investigating associations of the platelet-to-monocyte ratio (PMR), a novel hematological indicator of inflammatory responses with 30-day outcomes in patients with HBV-associated decompensated cirrhosis (HBV-DeCi).

**Methods:**

We recruited 329 patients with HBV-DeCi for this retrospective study and extracted baseline clinical data and laboratory characteristics from medical records. Univariate and multivariate analyses were performed to determine major factors influencing 30-day mortality. Receiver operating characteristic curve analysis was performed to compare the predictive values of prognostic markers.

**Results:**

During the 30-day follow-up period, 21 (6.4%) patients died. The PMR was significantly different between nonsurvivors and survivors. Lower PMR was found to be associated with an increased risk of 30-day mortality, and PMR (odds ratio: 1.011; 95% CI: 1.003–1.019; *P*=0.005) was found to be an independent predictor of 30-day mortality in patients with HBV-DeCi with a significant predictive value (AUC = 0.826, 95% CI: 0.781–0.865). The combination of PMR and MELD score could improve prognostic accuracy in these patients (AUC = 0.911, 95% CI: 0.876–0.940).

**Conclusions:**

Our results demonstrate that low PMR may be an independent predictor of 30-day mortality in patients with HBV-DeCi, and combined with the MELD score, it may be useful to complement other conventional measures to enable effective management of these patients.

## 1. Introduction

Hepatitis B virus (HBV) infection remains a major problem that endangers national health seriously in China. Many patients develop liver cirrhosis without receiving effective antiviral therapy [1]. Liver cirrhosis is a chronic, progressive, diffuse liver fibrosis with high morbidity and mortality. It is the 11^th^ most common cause of death in the world, causing approximately 1.03 million deaths each year [2, 3]. In China, nearly 3% of patients with compensated liver cirrhosis gradually progress to the decompensated stage characterized by overt clinical signs, such as ascites, rupture of esophageal and gastric varices, hepatic encephalopathy, and hepatorenal syndrome, which can lead to progressive multisystem organ failure. The survival rate of patients with HBV-related decompensated liver cirrhosis (HBV-DeCi) is not optimistic. The median survival is about 2 years in decompensated cirrhosis patients compared to more than 12 years in compensated cirrhosis patients, and the 5-year mortality rate is as high as 85% [4, 5]. Patients need to be hospitalized for multiple times and suffer heavy medical expenses. Liver transplantation is the most effective treatment method currently, which can significantly improve the survival rate. However, the shortage of liver sources, cost, and technical deficiencies limit its wide clinical application. Therefore, in order to accurately assess the severity of the patient's condition, it is urgent to find simple, objective, and effective biomarkers for the prognosis and disease monitoring of HBV-DeCi to improve the clinical management and survival rate of patients and reduce the economic burden.

Infection and increased systemic inflammation cause organ dysfunction and death in patients with HBV-DeCi. Previous studies provide support for the diagnostic and prognostic roles of a series of serological indicators in HBV-associated liver diseases, such as C-reactive protein (CRP) [6], CRP to albumin ratio (CAR) [7, 8], albumin-bilirubin score (ALBI) [9], aspartate aminotransferase to platelet ratio index (APRI) [10], fibrosis index based on four factors (FIB-4) [11], gamma-glutamyl transpeptidase-to-platelet ratio (GPR) [12], and gamma-glutamyl transpeptidase-to-albumin ratio (GAR) [13]. Monocytes, neutrophils, and lymphocytes are innate immune cells that can detect tissue damage, invade microorganisms, coordinate tissue healing, and eliminate infections. Inflammation can cause changes in the level of these blood cells. Therefore, the combination of these inflammatory indicators may be potential prognostic factors for predicting patients with decompensated liver cirrhosis.

In recent years, there is increasing evidence that neutrophil-to-lymphocyte ratio (NLR), lymphocyte-to-monocyte ratio (LMR), and platelet-to-lymphocyte ratio (PLR) are reliable inflammatory markers and prognostic indices in assessing the severity and mortality of various diseases such as cancers [14, 15], liver cirrhosis [16, 17], and myocardial infarction [18]. Due to their low cost and easy to obtain and interpret nature, these hematological ratios have been widely used in the laboratory testing. However, there are few related literature studies exploring the clinical significance of platelet-to-monocyte ratio (PMR) in HBV-DeCi. Therefore, the present study aimed at determining the role of PMR in predicting the 30-day mortality of HBV-DeCi patients to provide help for the future clinical management and prognosis.

## 2. Materials and Methods

### 2.1. Patients

Three hundred twenty-nine patients with HBV-DeCi who underwent treatment in the First Affiliated Hospital of Nanjing Medical University between January 5, 2017, and June 5, 2020, were retrospectively recruited. The inclusion criteria included an age of at least 18 years. The definition of HBV-related decompensated cirrhosis diagnosed standard: (1) positive for hepatitis B surface antigen (HBsAg) ≥ 6 months; (2) liver histology or ultrasonography and other imaging methods suggested cirrhosis; and (3) current or past ascites, rupture of esophageal and gastric varices, and hepatic encephalopathy complications [19]. Patients who met the following criteria were excluded: (1) malignancy, (2) hematological diseases, (3) history of other chronic liver disease (e.g., infection with hepatitis C virus and autoimmune hepatitis), (4) undergoing platelet transfusion or immunosuppressive therapy in the 3 months before the study period, and (5) missing data. The study and all its protocols were approved by the Institutional Ethics Committee of the First Affiliated Hospital of Nanjing Medical University.

### 2.2. Data Collection and Follow-Up

Relevant demographic and clinical data of the patients were obtained from medical records. Total protein, albumin, alanine aminotransferase (ALT), aspartate aminotransferase (AST), total bilirubin, creatinine, and blood urea nitrogen were measured in a Beckman Coulter AU5800 analyzer (Beckman Coulter, United States). Routine blood tests including hemoglobin levels, white blood cell (WBC), monocyte, and platelet (PLT) count were measured in a Sysmex XN series automated hematology analyzer (Sysmex, Japan). A Sysmex CS5100 automated blood coagulation analyzer (Sysmex, Japan) was used to determine the coagulation indices that included the international normalized ratio (INR). The PMR was defined as PLT divided by monocyte. Severity of liver disease was evaluated at the time of admission by the Model for End-Stage Liver Disease (MELD) score as previously described [20]. Patients were followed for 30 days to evaluate survival. Data on mortality were obtained from medical records or by telephone.

### 2.3. Statistical Analysis

All quantitative data are presented as the mean and standard deviation or median and interquartile range. Qualitative data are reported as count. Differences between quantitative variables were analyzed by the independent sample *t* test or Mann–Whitney *U* tests. The chi-squared test was used for qualitative variables. The correlation between the PMR and the MELD score was evaluated using Spearman's correlation test. In order to determine the risk factors of death in HBV-DeCi patients, univariate regression analysis was used. Then, covariates with *P* < 0.1 were included as candidate variables in the multivariate stepwise logistic regression analysis to identify independent predictors for the prognosis of HBV-DeCi patients. The receiver operating characteristic (ROC) curve was performed, and the area under the curve (AUC) was calculated to assess the prognostic value. DeLong's test was performed to compare the AUCs. Statistical analyses were performed using IBM SPSS 21.0 (SPSS Inc., Chicago, IL, USA) and MedCalc version 16.8.4 (MedCalc, Ostend, Belgium). Differences with bilateral *P* < 0.05 were considered statistically significant.

## 3. Results

### 3.1. Patient Characteristics

**Figure 1:**
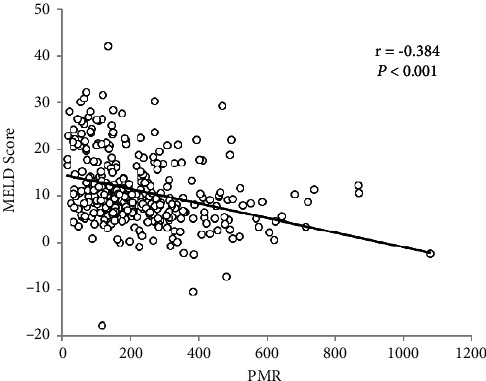
Scatterplot illustrating the correlation between PMR and MELD score in patients with HBV-DeCi.

After the exclusion criteria were applied, a total of 329 patients hospitalized with HBV-DeCi from January 2017 to June 2020 were enrolled in this retrospective study. Among the patients, 245 (74.5%) were male, and the median age was 51 years (range: 25–86 years). A significant negative correlation was found between the PMR and the MELD score (*r* = −0.384, *P* < 0.001) (Figure 1).

During the follow-up period, 308 patients survived and 21 died, giving a 30-day mortality rate of 6.4%. The demographic and laboratory parameters are compared between nonsurvivors and survivors in Table 1. The PMR was observed to be significantly lower in the nonsurvivors than that in survivors (median 82.43, IQR 45.66–112.01 vs. 194.04, 127.27–311.61, *P* < 0.001). Furthermore, compared to the survivors, the nonsurvivors also had a lower albumin level (*P*=0.02) and a much higher WBC, monocyte, neutrophil, total bilirubin level, MELD score, and INR (all *P* < 0.001). The other laboratory characteristics (i.e., gender ratio, median age, lymphocyte, hemoglobin, PLT, ALT, AST, and serum creatinine levels) were not significantly different between the two groups.

### 3.2. Independent Predictors of 30-Day Mortality in Patients with HBV-DeCi

**Figure 2:**
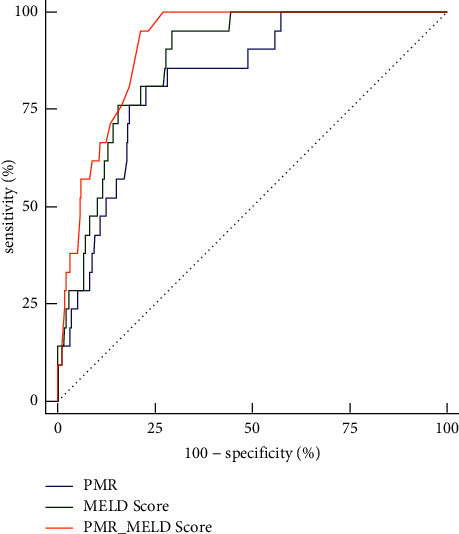
Receiver operating curves showing the relative prognostic performances of PMR and MELD score for the prediction of mortality in patients with HBV-DeCi.

Following the univariate analysis, variables less than 0.1 were entered into multivariate logistic regression. As shown in Table 2, the multivariate analysis revealed the MELD score and PMR to be independent predictors of poor outcomes after adjustments (*P* < 0.001, *P*=0.005). The ROC curves of the MELD score and PMR to predict mortality are illustrated in Figure 2. The cutoff value of the MELD score was found to be 12.21, which had a sensitivity of 95.24% and specificity of 70.45%. For PMR, the cutoff value was 118.62 with a sensitivity of 80.95% and specificity of 77.27%. The predictive powers of the MELD score and PMR for mortality were not significantly different, as indicated by the similar AUC values (0.874 for the MELD score vs. 0.826 for PMR; *Z* = 0.949, *P*=0.343). When the MELD score and PMR were analyzed in combination, the AUC (0.911) was slightly higher than that of the MELD score (*Z* = 1.741, *P*=0.082) and PMR (*Z* = 2.641, *P*=0.008), as were the specificity (95.24%) and the sensitivity (78.57%). The formula we used to combine the PMR and the MELD score was Logit *P* = 0.145 × MELD score − 0.011 × PMR − 3.360. The ROC curves and comparison of prognostic scores are shown in Table 3, respectively.

### 3.3. Baseline Characteristics and Factors Related to Platelet-to-Monocyte Blood Cell Ratio

HBV-DeCi patients were categorized into two groups: low-PMR group (PMR ≤ 118.62) and high-PMR group (PMR > 118.62), according to the cutoff value obtained by the ROC analysis. Clinical data and laboratory findings are demonstrated in Table 4. There was no significant difference in age, hemoglobin, ALT, or AST. Low PMR was found to be associated with higher WBC, LY, MO, NE, total bilirubin, serum creatinine, MELD score, INR, and mortality rate but lower PLT and albumin (all *P* < 0.05). Our findings indicated that a decreased PMR level at admission was followed by an increased 30-day mortality rate, increasing from 1.7% in the high-PMR group to 24.3% in the low-PMR group.

## 4. Discussion

This study firstly identified that the PMR was an independent predictor for 30-day mortality in HBV-DeCi patients, and the combination of the PMR and the MELD score could improve prognostic accuracy in these patients. Furthermore, the results also displayed that there was a significant inverse correlation between these two indicators.

Currently, the MELD score has been an ideal scoring system for assessing the severity of end-stage liver disease and widely used for the evaluation of organ allocation in liver transplant patients [21]. Compared with the Child-Pugh scoring system, the MELD model can assess the severity more objectively and accurately due to containing no subjective indicators such as ascites and hepatic encephalopathy, which may vary according to the physicians' judgement. However, the MELD score does not consider all factors that may affect the prognosis, such as inflammation. The creatinine level in the model is easily influenced by hemodynamics and diuretics. Besides, many factors such as starvation and systemic infections can affect serum bilirubin. Accounting for these limitations, approximately 15%–20% of liver transplantation candidates are not well served by the MELD score [22]. In this study, we find that nonsurvivors have lower PMR levels than survivors. In addition, PMR is independently related to the adverse outcome of HBV-DeCi patients and has similar predictive power to the MELD score. The unique advantages of the laboratory-based PMR are its objectivity, time-saving nature, and noninvasive interpretation, which are much easier to obtain and cheaper than the MELD score. Of note, the combination of PMR and MELD score further improves the prognostic accuracy of adverse outcomes compared with PMR or MELD alone. Our findings demonstrate that PMR can be used as an independent predictor of 30-day mortality in patients with HBV-DeCi, and when it is combined with the MELD score, it may perform better to enable effective management of these patients.

The underlying mechanism between PMR and prognosis in patients with HBV-DeCi needs to be illustrated. Our results indicate that the decrease of the PMR level in nonsurvivors is mainly due to the elevated number of monocytes and slightly increased number of PLT compared with surviving patients. It is generally known that inflammation plays a key role in the progression of liver cirrhosis and is linked to adverse outcomes [23, 24]. Monocytes can differentiate into different cell groups and serve an important function in the innate and acquired immune response. The inflammatory response can trigger the release of monocytes from bone marrow into the peripheral blood [25]. Accumulated evidence shows that patients with liver cirrhosis have mononucleosis, which is positively correlated with disease progression [26]. Therefore, the markedly increase in monocytes that we observed among nonsurvivors may indicate persistent inflammation, which lead to the poor prognosis. However, the relationship between changes in the number, phenotype, and function of monocytes and their subpopulations and disease progression during pathogen infection, especially in severe chronic HBV infection, remains unclear.

Among the 329 patients in the present study, 246 (74.8%) have thrombocytopenia (PLT < 100 × 10^9^/L). Thrombocytopenia, common in liver cirrhosis, is a multifactorial condition involving both impaired thrombopoiesis and increased platelet clearance [27, 28]. The pathophysiology of thrombocytopenia in patients with cirrhosis has long been associated with the hypothesis of hypersplenism, in which splenic congestion and portal hypertension cause the mononuclear-macrophage system to phagocytose platelets, resulting in thrombocytopenia and functional defects [29]. The discovery of thrombopoietin (TPO) illustrates another central mechanism. TPO is mainly produced by the liver and is decreased when hepatocytes are seriously damaged. This can lead to decreased platelet production in the bone marrow, resulting in thrombocytopenia in patients with cirrhosis [30]. In addition, immune complexes are produced that destroy bone marrow megakaryocytes, leading to a block in platelet production.

According to the already performed surveys, PLT is reported to be significantly associated with survival in patients with liver cirrhosis and plays a vital role in coordinating inflammatory and immune responses [31]. However, according to the multivariate analysis in our study, it was found that neither monocytes nor PLT were independent predictors of mortality. This difference may have arisen because PLT and monocytes are individual parameters, which can be altered by several variables such as dehydration, overhydration, or blood specimen handling. In contrast, the PMR is much more stable as a ratio. It can be quickly calculated at the patient's bedside using a simple formula and easy to be applied for daily clinical practice. Furthermore, we also found a significant association between the low PMR and the high MELD score as well as 30-day mortality, suggesting that the lower PMR may be predictive of the severity and progression of liver injury and affect the short-term prognosis of patients with HBV-DeCi, while the exact mechanism of PMR to explain its association with prognosis and correlation with the MELD score remains elusive.

A few limitations in our study need to be considered. First, our single-center study is designed retrospectively in nature, which may have led to a selection bias. The findings need to be verified by prospective and multicenter studies with larger sample numbers. Second, only 21 people died in the entire cohort, which made the study underpowered. Third, we fail to give virological data and inflammatory biomarkers, such as viral loads or CRP, in our study, which might be useful in elucidating the association between PMR and adverse outcome. We focused on DeCi mainly caused by HBV infection. Whether these findings are applicable to other etiologies of DeCi needs further study.

## 5. Conclusions

In summary, this study concludes that the PMR can be utilized as an independent predictor for 30-day mortality in HBV-DeCi patients. A combination of PMR and MELD score further augmented the predicting power. Due to its time-saving and noninvasive nature, the PMR may be a useful supplement to standard approaches to enable effective management of these HBV-DeCi patients.

## Figures and Tables

**Table 1:** Patient characteristics at the baseline.

Variables	All patients (*n* = 329)	Survivors (*n* = 308)	Nonsurvivors (*n* = 21)	*P*
Gender (female/male)	84/245	80/228	4/17	0.483
Age (years)	51 (44–60)	51 (43–59)	51 (46–67)	0.088
WBC (×10^9^/L)	4.19 (2.91–6.72)	3.97 (2.82–6.50)	6.78 (5.44–8.46)	<0.001
Lymphocyte (×10^9^/L)	0.87 (0.54–1.44)	0.86 (0.53–1.43)	1.21 (0.62–1.67)	0.489
Monocyte (×10^9^/L)	0.34 (0.23–0.61)	0.32 (0.22–0.55)	0.77 (0.45–1.11)	<0.001
Neutrophil (×10^9^/L)	2.66 (1.70–4.73)	2.53 (1.67–4.38)	5.34 (3.65–6.89)	<0.001
Hemoglobin (g/L)	101.55 ± 25.67	101.95 ± 25.59	95.71 ± 26.80	0.282
PLT (×10^9^/L)	63.0 (41.0–101.5)	60.5 (41.0–100.5)	65.0 (37.5–105.0)	0.523
PMR	184.62 (115.27–300.55)	194.04 (127.27–311.61)	82.43 (45.66–112.01)	<0.001
Albumin (g/L)	30.7 ± 6.2	31.0 ± 6.2	27.7 ± 5.3	0.020
ALT (U/L)	30.8 (19.2–63.7)	29.0 (18.8–61.2)	50.6 (26.5–107.6)	0.475
AST (U/L)	45.3 (29.1–80.6)	42.8 (28.8–78.4)	77.8 (52.3–165.2)	0.682
Total bilirubin (*μ*mol/L)	28.2 (17.2–55.1)	27.4 (16.7–49.7)	231.6 (52.0–428.3)	<0.001
Creatinine (*μ*mol/L)	64.7 (53.2–76.6)	64.3 (52.7–75.3)	80.9 (55.9–122.0)	0.308
MELD score	9.69 (6.36–14.62)	9.09 (6.09–13.28)	21.10 (16.00–26.70)	<0.001
INR	1.38 (1.26–1.59)	1.38 (1.25–1.58)	1.81 (1.41–2.55)	<0.001

Data are expressed as number, mean ± standard deviation, or median (interquartile range).

**Table 2:** Logistic regression analysis to identify risk factors associated with mortality in patients with HBV-DeCi.

Variables	Univariate		Multivariate	
	Odds ratio (95% CI)	*P*	Odds ratio (95% CI)	*P*
Age (years)	1.031 (0.995–1.068)	0.089		
WBC (×10^9^/L)	1.136 (1.054–1.223)	0.001		
LY (×10^9^/L)	1.188 (0.729–1.938)	0.489		
MO (×10^9^/L)	3.528 (1.776–7.009)	<0.001		
NE (×10^9^/L)	1.160 (1.064–1.265)	0.001		
Hemoglobin (g/L)	1.010 (0.992–1.028)	0.282		
PLT (×10^9^/L)	1.003 (0.995–1.011)	0.523		
PMR	1.016 (1.008–1.024)	<0.001	1.011 (1.003–1.019)	0.005
Albumin (g/L)	1.093 (1.013–1.181)	0.022		
ALT (U/L)	1.000 (0.999–1.002)	0.496		
AST (U/L)	1.000 (0.999–1.002)	0.691		
Total bilirubin (*μ*mol/L)	1.007 (1.004–1.009)	<0.001		
Serum creatinine (*μ*mol/L)	1.002 (0.998–1.006)	0.327		
MELD score	1.194 (1.120–1.273)	<0.001	1.156 (1.081–1.236)	<0.001
INR	5.088 (2.401–10.782)	<0.001		

Abbreviations: CI, confidence interval.

**Table 3:** Comparison of prognostic scores in predicting 30-day mortality.

Prognostic scores	AUC	95% CI	Cut-off points	Sensitivity (%)	Specificity (%)	PLV	NLV
PMR	0.826	0.781–0.865	118.62	80.95	77.27	3.56	0.25
MELD score	0.874	0.834–0.908	12.21	95.24	70.45	3.22	0.068
PMR + MELD score	0.911	0.876–0.940	0.05	95.24	78.57	4.44	0.061

PLV, positive likelihood ratio; NLV, negative likelihood ratio.

**Table 4:** Clinical data according to the value of platelet-to-monocyte ratio.

Variables	Low group (PMR ≤ 118.62, *n* = 87)	High group (PMR > 118.62, *n* = 242)	*P*
Gender (female/male)	12/75	72/170	0.003
Age (years)	49 (42–59)	51 (45–60)	0.226
WBC (×10^9^/L)	7.09 (4.38–11.01)	3.65 (2.58–5.50)	<0.001
Lymphocyte (×10^9^/L)	1.15 (0.61–1.69)	0.84 (0.53–1.37)	0.005
Monocyte (×10^9^/L)	0.71 (0.39–1.17)	0.28 (0.20–0.45)	<0.001
Neutrophil (×10^9^/L)	5.24 (3.12–7.75)	2.22 (1.59–3.59)	<0.001
Hemoglobin (g/L)	100.05 ± 28.81	102.10 ± 24.49	0.525
PLT (×10^9^/L)	50.0 (31.0–73.0)	66.5 (45.0–119.0)	<0.001
Albumin (g/L)	28.75 ± 5.86	31.46 ± 6.11	<0.001
ALT (U/L)	42.9 (20.2–75.5)	28.15 (18.55–58.53)	0.073
AST (U/L)	54.4 (33.8–97.4)	41.7 (28.80–76.08)	0.051
Total bilirubin (*μ*mol/L)	46.10 (23.50–139.40)	25.00 (15.88–48.40)	<0.001
Serum creatinine (*μ*mol/L)	69.30 (57.10–95.20)	63.05 (51.75–73.90)	0.001
MELD score	13.37 (9.02–21.74)	8.69 (5.69–12.00)	<0.001
INR	1.50 (1.27–1.87)	1.37 (1.24–1.53)	<0.001
30-day mortality (yes/no)	17/70 (24.3%)	4/238 (1.7%)	<0.001

Data are expressed as number, mean ± standard deviation, or median (interquartile range).

## Data Availability

The datasets are available from the corresponding author upon reasonable request.

## References

[1] Ginès P., Krag A., Abraldes J. G., Solà E., Fabrellas N., Kamath P. S. (2021). Liver cirrhosis. *The Lancet*.

[2] Asrani S. K., Devarbhavi H., Eaton J., Kamath P. S. (2019). Burden of liver diseases in the world. *Journal of Hepatology*.

[3] Roth G. A., Abate D., Abate K. H. (2018). Global, regional, and national age-sex-specific mortality for 282 causes of death in 195 countries and territories, 1980-2017: a systematic analysis for the Global Burden of Disease Study 2017. *Lancet (London, England)*.

[4] D’Amico G. (2014). The clinical course of cirrhosis. Population based studies and the need of personalized medicine. *Journal of Hepatology*.

[5] Angeli P., Bernardi M., Villanueva C. (2018). Electronic address: easloffice@easloffice.eu; European Association for the Study of the Liver. EASL Clinical Practice Guidelines for the management of patients with decompensated cirrhosis. *Journal of Hepatology*.

[6] Wu J., Wu Q., Wu M., Mao W. (2019). Serum cystatin C predicts mortality in HBV-related decompensated cirrhosis. *BioMed Research International*.

[7] Huang S. S., Xie D. M., Cai Y. J. (2017). C-reactive protein-to-albumin ratio is a predictor of hepatitis B virus related decompensated cirrhosis: time-dependent receiver operating characteristics and decision curve analysis. *European Journal of Gastroenterology and Hepatology*.

[8] Wang C. J., Wu J. P., Zhou W. Q., Mao W. L., Huang H. B. (2019). The C-reactive protein/albumin ratio as a predictor of mortality in patients with HBV-related decompensated cirrhosis. *Clinical Laboratory*.

[9] Qi X. T. (2018). Albumin-bilirubin score predicts short-term mortality in patients with hepatitis B virus-related decompensated cirrhosis. *Clinical Laboratory*.

[10] Mao W., Sun Q., Fan J., Lin S., Ye B. (2016). AST to platelet ratio index predicts mortality in hospitalized patients with hepatitis B-related decompensated cirrhosis. *Medicine*.

[11] Kim M. N., Lee J. H., Chon Y. E., Ha Y., Hwang S. G. (2020). Fibrosis-4, aspartate transaminase-to-platelet ratio index, and gamma-glutamyl transpeptidase-to-platelet ratio for risk assessment of hepatocellular carcinoma in chronic hepatitis B patients: comparison with liver biopsy. *European Journal of Gastroenterology and Hepatology*.

[12] Yu K., Du Z., Li Q. (2019). Comparison of non-invasive models for predicting liver damage in chronic hepatitis B patients. *Gastroenterología y Hepatología*.

[13] Li Y., Chen Y., Zhao Y. (2014). The diagnostic value of the FIB-4 index for staging hepatitis B-related fibrosis: a meta-analysis. *PLoS One*.

[14] Liu D., Huang Y., Li L., Song J., Zhang L., Li W. (2017). High neutrophil-to-lymphocyte ratios confer poor prognoses in patients with small cell lung cancer. *BMC Cancer*.

[15] Hu G., Liu G., Ma J. Y., Hu R. J. (2018). Lymphocyte-to-monocyte ratio in esophageal squamous cell carcinoma prognosis. *Clinica Chimica Acta*.

[16] Cai Y. J., Dong J. J., Dong J. Z. (2017). A nomogram for predicting prognostic value of inflammatory response biomarkers in decompensated cirrhotic patients without acute-on-chronic liver failure. *Alimentary Pharmacology & Therapeutics*.

[17] Piotrowski D., Sączewska-Piotrowska A., Jaroszewicz J., Boroń-Kaczmarska A. (2020). Lymphocyte-to-monocyte ratio as the best simple predictor of bacterial infection in patients with liver cirrhosis. *International Journal of Environmental Research and Public Health*.

[18] Li L., Ma Y., Geng X. B. (2021). Platelet-to-lymphocyte ratio relates to poor prognosis in elderly patients with acute myocardial infarction. *Aging Clinical and Experimental Research*.

[19] Yoshiji H., Nagoshi S., Akahane T. (2021). Evidence-based clinical practice guidelines for Liver Cirrhosis 2020. *Journal of Gastroenterology*.

[20] Freeman R. B., Wiesner R. H., Harper A. (2002). The new liver allocation system: moving toward evidence-based transplantation policy. *Liver Transplantation*.

[21] Huo T. I., Lee S. D., Lin H. C. (2008). Selecting an optimal prognostic system for liver cirrhosis: the model for end-stage liver disease and beyond. *Liver International*.

[22] Kamath P. S., Kim W. R. (2007). The model for end-stage liver disease (MELD). *Hepatology*.

[23] Clària J., Stauber R. E., Coenraad M. J. (2016). Systemic inflammation in decompensated cirrhosis: characterization and role in acute-on-chronic liver failure. *Hepatology*.

[24] Albillos A., Lario M., Álvarez-Mon M. (2014). Cirrhosis-associated immune dysfunction: distinctive features and clinical relevance. *Journal of Hepatology*.

[25] Shi C., Pamer E. G. (2011). Monocyte recruitment during infection and inflammation. *Nature Reviews Immunology*.

[26] Jamil Z., Durrani A. A. (2018). Assessing the outcome of patients with liver cirrhosis during hospital stay: a comparison of lymphocyte/monocyte ratio with MELD and Child-Pugh scores. *Turkish Journal of Gastroenterology: The Official Journal of Turkish Society of Gastroenterology*.

[27] Peck-Radosavljevic M. (2017). Thrombocytopenia in chronic liver disease. *Liver International*.

[28] Pradella P., Bonetto S., Turchetto S. (2011). Platelet production and destruction in liver cirrhosis. *Journal of Hepatology*.

[29] Lee E. J., Lee A. I. (2016). *Primary Care: Clinics in Office Practice*.

[30] van Dievoet M. A., Eeckhoudt S., Stephenne X. (2020). Primary hemostasis in chronic liver disease and cirrhosis: what did we learn over the past decade?. *International Journal of Molecular Sciences*.

[31] Storey R., Thomas M. (2015). The role of platelets in inflammation. *Thrombosis and Haemostasis*.

